# Unusually large ephedrine-induced blood pressure increases due to cardiac sympathetic denervation supersensitivity in a patient with Parkinson’s disease

**DOI:** 10.1186/s40981-018-0181-2

**Published:** 2018-06-06

**Authors:** Toru Shirai, Atsuhiro Kitaura, Keiji Uehara, Tomohisa Uchida, Masaki Fuyuta, Tatushige Iwamoto, Kenji Hiramatsu, Shinichi Nakao

**Affiliations:** Department of Anesthesiology, Kindai University Faculty of Medicine, 377-2, Ohno-Higashi, Osakasayama, Osaka 589-8511 Japan

**Keywords:** Cardiac sympathetic denervation, Denervation supersensitivity, Ephedrine, Metaiodobenzylguanidine, Parkinson’s disease

## Abstract

**Background:**

Parkinson’s disease (PD) patients often suffer from cardiac sympathetic denervation, a hallmark of which is orthostatic hypotension. Denervation supersensitivity to sympathomimetic drugs is also seen in such patients, and this phenomenon is important and can be sometimes dangerous.

**Case presentation:**

A 65-year-old male underwent gastrojejunostomy. The patient had severe PD and did not exhibit metaiodobenzylguanidine (MIBG) accumulation in his heart, which was indicative of cardiac sympathetic nerve denervation. When 8 mg of ephedrine was administered intravenously, an unexpectedly large increase in blood pressure was observed. The phenomenon recurred when 4 mg of ephedrine was administered again, and nicardipine was required to suppress the patient’s blood pressure.

**Conclusions:**

Denervation supersensitivity is not as well recognized as other complications seen in PD patients, but anesthesiologists should be aware of it because sympathomimetic drugs can have excessively strong effects in patients with the condition.

## Background

Parkinson’s disease (PD) is a common and complex neurodegenerative disorder. The pathological hallmark of PD is loss of dopaminergic neurons within the substantia nigra pars compacta. The resultant dopaminergic deficiency within the basal ganglia results in movement disorders, such as bradykinesia (i.e., slowness during the initiation of voluntary movements with a progressive reduction in the speed and amplitude of repetitive actions), muscle rigidity, resting tremors, and postural instability [1]. In addition to these motor features, cognitive impairment, psychiatric symptoms, and autonomic dysfunction are also recognized in PD patients [1]. Of these, orthostatic hypotension, an autonomic nerve disorder, is frequently seen in PD patients, and it has been demonstrated to be caused by cardiac sympathetic nerve denervation, which results in sympathetic neurotransmitter noradrenaline deficiency in the heart and blood vessels [2]. On the other hand, there have only been a few reports about excessive cardiovascular responses to sympathomimetic drugs in PD patients due to denervation supersensitivity [3, 4]. This phenomenon is very important and can be sometimes dangerous but seems to be rarely recognized and poorly understood.

Here, we present a case of suspected supersensitivity to ephedrine in a patient with PD.

## Case presentation

A 65-year-old male (160 cm, 65 kg) with gastric outlet obstruction due to a stomach ulcer scar was scheduled for gastrojejunostomy. His medical history included PD and hypertension. He had suffered from PD since he was 58 years old, and had then orthostatic hypotension, occasional tremors in both upper limbs, slow movement, and muscle rigidity. Although supine hypertension (SH) is recognized as a feature of cardiovascular autonomic failure that often accompanies orthostatic hypotension in patients with PD, the SH was not documented in the present case because the tilt test, which can detect SH [5], was not performed. His PD symptom progression was scored as stage 4 according to the Hoehn and Yahr scale [6], which consists of five stages of 1 (light) to 5 (severe). Stage 4 is defined as patients with severely disabling disease, but still able to walk or stand unassisted. He was orally taking 20 mg of nicardipine for hypertension, and 400 mg of levodopa, 10.8 mg of carbidopa, 100 mg of entacapone, 25 mg of zonisamide, and 36 mg of rotigotine for PD. His preoperative blood pressure and heart rate were in good control with around 130/80 and 60–80 beats per min, respectively. His preoperative electrocardiograph showed a normal sinus rhythm with no ST-T changes, and echocardiography revealed no abnormalities. However, severely decreased ^123^I-metaiodobenzylguanidine (MIBG) accumulation in the heart was observed. The early heart-to-mediastinum (H/M) ratio, which was obtained 15 min after the injection of ^123^I-MIBG, was 1.305 (normal range > 2.0); the late H/M ratio, which was obtained 4 h after the injection of ^123^I-MIBG, was 1.099 (normal range > 2.0); and the washout rate (WOR) was 73.4% (normal range 9–20%). Information on the distribution of neurons and function of the re-uptake pathway is provided by the H/M ratio, while the WOR provides information on the sympathetic drive [7].

**Fig. 1:**
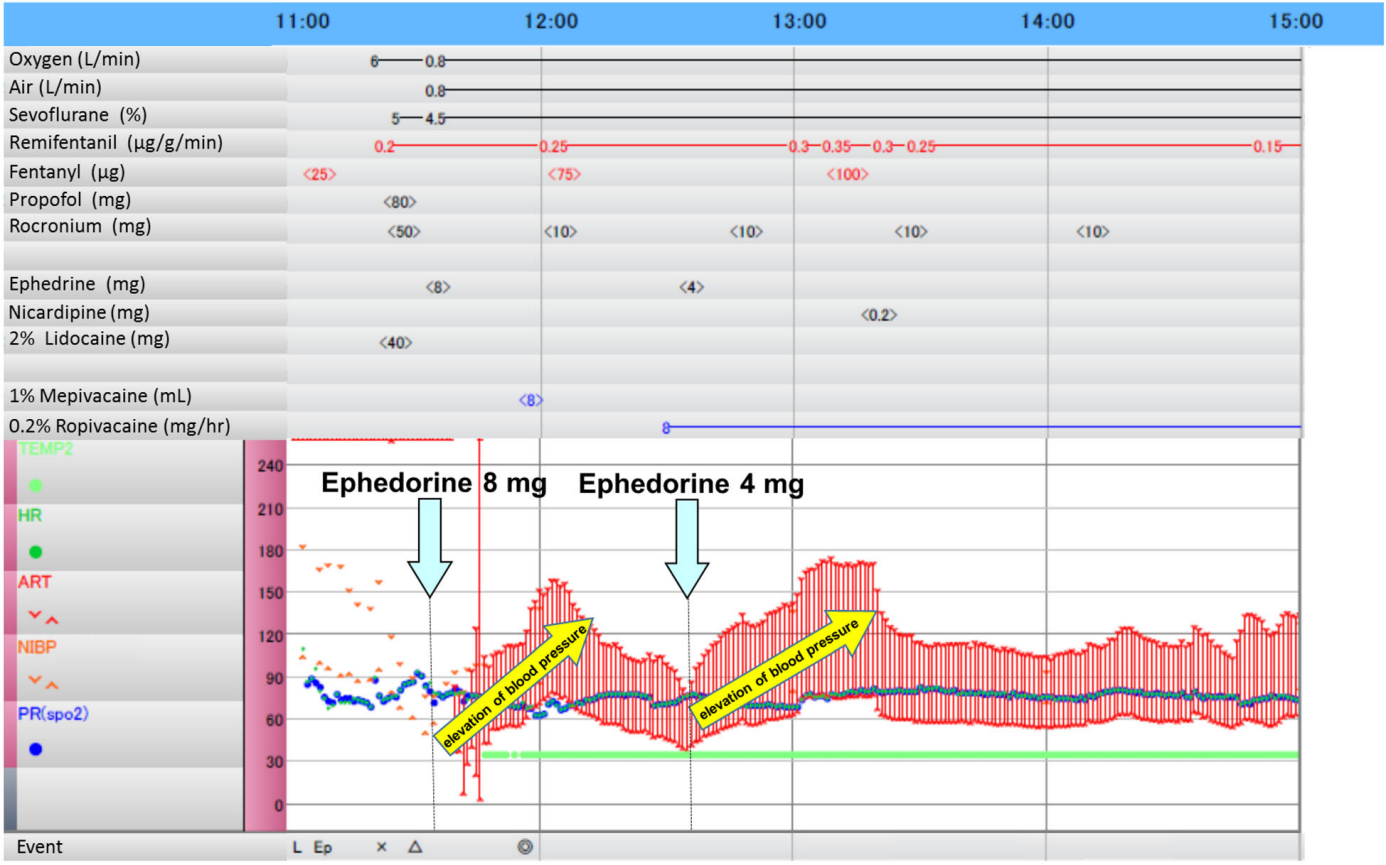
The patient’s anesthesia chart. Small amounts of ephedrine (8 or 4 mg) reproducibly induced large increases in blood pressure, and treatment with nicardipine, a calcium channel blocker, was required to suppress the second blood pressure increase

In the operating room, after the placement of standard monitors, a Touhy needle was inserted into the epidural space at Th_10–11_, and an epidural catheter was inserted 5 cm into the epidural space. Then, 3 ml of 1% mepivacaine was injected through the catheter with no motor block. General anesthesia was induced with 80 mg propofol and remifentanil, and the trachea was intubated with the aid of 60 mg rocuronium. Anesthesia was maintained with an oxygen/air mixture and 4.5–5% desflurane. Eight milliliters of 1% mepivacaine was injected into the epidural space 10 min before the operation, and a continuous infusion of 188 ml of 0.2% ropivacaine and 0.6 mg fentanyl into the epidural space was started at a rate of 4 ml/h. Eight milligrams of ephedrine was administered intravenously when the patient’s blood pressure decreased, and an unexpectedly large increase in blood pressure was observed. This phenomenon recurred when 4 mg of ephedrine was administered again, but this time, nicardipine was required to suppress the patient’s blood pressure (Fig. 1). Interestingly, his heart rate was not affected by ephedrine (Fig. 1). At this time, we considered that denervation supersensitivity to ephedrine had been induced and stopped using catecholamines. After the completion of the operation, the patient was extubated. His postoperative course was uneventful.

## Discussion

We reported a case in which ephedrine unexpectedly caused a marked increase in blood pressure in a patient with PD, whose cardiac sympathetic nerves were almost completely denervated. Cardiac sympathetic denervation can be diagnosed based on a reduction in or loss of MIBG accumulation in the heart. MIBG is an analog of noradrenaline, which is taken up by adrenergic neurons in a similar fashion to noradrenaline, but does not undergo intracellular metabolism or exhibit physiological activity [2]. The early H/M ratio reflects the integrity of presynaptic nerve terminals and their uptake functions. The late H/M ratio provides information on neuronal functions from uptake mechanisms to the release of neurotransmitters from the storage vesicles located at nerve terminals. The MIBG WOR is an index of the degree of sympathetic drive [7]. In the present case, the patient had severely low H/M ratios both in the early phase and in the late phase and a markedly high washout rate, all of which were indicative of cardiac sympathetic denervation.

Orthostatic hypotension, which can be caused by cardiac sympathetic denervation, is easy to detect and, consequently, is well recognized in patients with PD. However, denervation supersensitivity does not appear to be as well recognized as orthostatic hypotension, probably because it can only be detected when sympathomimetic drugs are administered. In addition, it is easy to overlook this phenomenon because there are so many causes of blood pressure elevations and/or heart rate increases. Miyamoto et al. reported a case of ventricular tachycardia (VT) in a PD patient, which occurred after noradrenaline was administered at a rate of 0.0 8–0.25 μg/kg/min to treat hypotension due to septic shock [4]. The VT subsided when vasopressin was administered instead of noradrenaline. Furthermore, Nakamura et al. demonstrated that administering dobutamine at a rate of 4 μg/kg/min increased both systolic pressure and cardiac contractility more in PD patients than in controls, and this hyperdynamic response was significantly correlated with reduced H/M ratios [8]. In the current case, the patient suffered from orthostatic hypotension preoperatively and exhibited severely reduced cardiac MIBG accumulation, but his denervation supersensitivity was not recognized. However, the administration of 8 mg of ephedrine induced an unusually marked increase in the patient’s blood pressure when it was first administered. A second dose of ephedrine (4 mg) also induced a marked increase in blood pressure. It is true that ephedrine sometimes markedly increases either heart rate or blood pressure, but in the present case, the degree of the blood pressure increases induced by small amounts of ephedrine was unusual (to the extent that nicardipine was required). Thus, we consider that the most plausible explanation for the observed increases in blood pressure was the patient’s cardiac denervation supersensitivity. As the mechanisms of denervation supersensitivity are ascribed to the absence of norepinephrine uptake and/or to increased density of β-adrenergic receptors in post-synaptic membranes [9], drugs which do not have β-adrenergic stimulant effects, such as phenylephrine and vasopressin, would be better and safer. Interestingly, the blood pressure elevations by ephedrine were gradual and continued for a while in the present case, and without nicardipine administration after the second ephedrine administration, the blood pressure would have continued to increase further (Fig. 1). We cannot clarify the reason of this phenomenon, but it may have taken time to activate intracellular signal transduction system in the heart cells. In the report of Miyamoto et al., VT also happened 2 h after the start of noradrenaline infusion [4]. More interestingly, the patient’s heart rate did not change markedly after the administration of ephedrine, even though ephedrine has β-adrenergic as well as α-adrenergic effects, probably because any heart rate increase was suppressed by a reflex secondary to the rise in blood pressure. Similarly, Nakamura et al. reported that dobutamine did not result in greater heart rate increases in PD patients than in controls, in spite of the fact that it caused a marked elevation of blood pressure [8].

## Conclusions

We encountered a case in which ephedrine induced an unusually large increase in blood pressure in a patient with PD, whose cardiac sympathetic nerves were almost completely denervated. We need to know that PD patients often suffer from cardiac sympathetic nerve denervation and cardiac denervation supersensitivity to sympathomimetic drugs.

## Abbreviations

MIBG: Metaiodobenzylguanidine
PD: Parkinson’s disease
VT: Ventricular tachycardia
